# Environmental Factors, Developmental Genes and Oxidative Stress Determine Inter-Species Variability in Seed Longevity in Salicaceae

**DOI:** 10.3390/plants14182861

**Published:** 2025-09-13

**Authors:** Xiaoyin Zhang, Qin Ai, Xiaojian Hu, Liang Lin, Xiangyun Yang, Hugh W. Pritchard, Jie Cai, Huajie He, Hongying Chen

**Affiliations:** Germplasm Bank of Wild Species, Kunming Institute of Botany, Chinese Academy of Sciences, Kunming 650201, China; zhangxiaoyin@mail.kib.ac.cn (X.Z.); aiqin@mail.kib.ac.cn (Q.A.); huxiaojian@mail.kib.ac.cn (X.H.); linliang@mail.kib.ac.cn (L.L.); yxy@mail.kib.ac.cn (X.Y.); hwp@mail.kib.ac.cn (H.W.P.); j.cai@mail.kib.ac.cn (J.C.)

**Keywords:** short-lived seeds, cryopreservation, seed traits, seed storage, environmental factors

## Abstract

Dry seed longevity varies considerably among species, but little is known about its relation with the climate and the molecular mechanisms that determine seed lifespan. Salicaceae species, with more than 620 species worldwide, are known to produce short-lived seeds, making them particularly good models to explore ageing processes in the glassy state rather than under accelerated ageing. We compared seed lifespan for 13 species of *Salix* and *Populus* across a broad geographical range (up to 2200 m a.s.l.). High-quality seeds were obtained by optimizing collection time (just before capsule dehiscence) and post-harvest handling (i.e., the use of negative pressure to remove seed hairs). At optimal moisture contents (MCs) between 6 and 9%, most species seeds demonstrated minimal decreases in viability after storage at −20 °C or in liquid nitrogen for 3 years. Dry room (15% RH, 15 °C) storage differentiated between species’ seed lifespans (P_50_s) of c. 150 to >1200 d. Unlike *Salix*, *Populus* species from warm wet environments tended to produce longer-lived seeds in dry storage. Based on transcriptome data on *Populus davidiana* (longer-lived) and *Populus euphratica* (shorter-lived), we revealed high correlations between late seed maturation genes, such as 60% of *HSP* and 67% of *LEA* genes showed higher expression in *P. davidiana* seeds, while 70% of *WRKY* transcription factors showed significantly higher expression in *P. euphratica* seeds. For these two species, genes related to oxidative stress might be the most important contributor to different seed longevity in the dry glassy state.

## 1. Introduction

Seed longevity, as a multifaceted adaptive trait, governs survival plasticity across both natural and anthropogenic environments, encompassing in situ preservation within soil seed banks and ex situ banking for conservation and accelerated ageing trials [1]. Seed longevity determines the scheduling of viability retesting and informs regeneration or recollection protocols, making interspecific variation in longevity critical for optimizing ex situ conservation management and agricultural practices [2]. As it is impossible to obtain seed longevity by experimentation on all plants, some general predictive models have been developed using co-correlates of longevity, mainly for large data sets for species’ seeds held under one condition [1,3]. For example, seed longevity in 195 wild species was compared during controlled accelerated ageing at 45 °C or 60 °C and 60% relative humidity (RH), revealing phylogenetically conserved longevity patterns [4]. In particular, endospermic seeds tended to exhibit reduced longevity compared to non-endospermic seeds, and seeds originating from hot, dry environments lived longer than those from cool, wet habitats. A phylogenetic biogeographic analysis with controlled accelerated ageing further revealed that European-origin accessions from temperate climates showed accelerated viability loss, whereas South Asian and Australian accessions from tropical ecosystems maintained optimal longevity [5]. Other investigations of orthodox seeds under cool (5 °C), dry (3.5–9.5% moisture content [MC]) storage (42 species) and cold storage (−18 °C, 4–8% MC) identified taxon-specific degradation kinetics, for example, with Apiaceae and Brassicaceae displaying diminished longevity in contrast to Malvaceae and Chenopodiaceae that maintained viability beyond 23 years [6].

During seed drying to low RH, cellular viscosity increases and the cytoplasm transforms into a glassy state [7]. Such glasses have slow molecular mobility, slower reactant mobility (O_2_, H_2_O) and degradation reaction rates. Importantly, the glass transition temperature is dependent on seed moisture content and accelerated seed ageing under elevated temperature and moisture means the seeds are likely in a non-glassy (rubbery) state [1]. Many longevity studies have employed such elevated relative humidity (RH > 60%) and thermal stress (35–50 °C) conditions. Whilst such studies have revealed the roles of programmed cell death and decline in antioxidant capacity in viability loss [8], extrapolation of ageing characteristics between distinct thermodynamic regimes (e.g., glassy vs. rubbery states) remains problematic [9], as the fundamental mechanisms of ageing diverge [3,10,11,12]. For example, reactive oxygen species (ROS) produced through auto-oxidative process occurs at a slow rate under dry (glassy) ageing condition [11]. Accumulation of ROS will result in protein, lipid and DNA damage. However, the dry seeds’ DNA damage can be repaired during storage at higher moisture contents and during early imbibition by major pathways, including base and nucleotide excision repair (BER, NER) and the repair of chromosomal breaks by non-homologous end joining (NHEJ) and homologous recombination (HR) [Waterworth, 2024] [13]. In addition, DNA damage signalling kinases ATAXIA TELANGIECTASIA MUTATED (ATM) and ATM AND RAD3-RELATED (ATR) orchestrate plant cellular response to DNA damage [13]. Thus, the best predictors for seed longevity under glassy states (e.g., seed bank dry rooms and freezers) will depend on the development of specific models and an understanding of mechanisms determined under such conditions [11].

The rate of seed viability loss [14] can be characterized as a linear decrease in germination (in probit units) with storage time. The resulting slope describes the standard deviation (also known as σ) of the distribution of seed deaths over time (also known as σ) and the intercept of the viability loss line at time zero provides an estimate of the initial viability, Ki, of the seed lot. Therefore, *v* = *Ki* − *p* ⁄σ where *v* is the viability, in probit units, after *p* days of seed storage. The period that seeds remain viable depends on σ (longevity or days to lose one probit of viability), which varies with both moisture content and temperature and as described by the seed viability equation [15]:

log σ = *K_E_* − *C_W_* log*MC* − *C_H_T* − *C_Q_T*^2^

where *C_H_* and *C_Q_* are constants associated with temperature and *K_E_* and *C_W_* are constants associated with moisture, where MC is the moisture content (% fresh weight basis) and T is temperature (°C). Whilst *K_E_* and *C_W_* tend to vary between species, *C_H_* and *C_Q_* are thought to represent universal temperature constants for species [16]. One value of analyzing viability loss responses in this way is being able to estimate the time taken for seeds to lose 50% of viability (P_50_), enabling inter-species comparisons in seed ageing performance when the seeds are being held under identical environmental conditions [4].

Dry seeds of many Salicaceae species, including the genera *Populus* L. (poplars) and *Salix* L. (willows), are reported to be short-lived [17,18], and potentially have relatively short P_50_s, making them ideal material to explore ageing process in the glassy state. Salicaceae includes many species of high economic and ecological value and conservation concern [19]. For example, *Populus nigra* is considered to be threatened species in Europe, and *Populus shanxiensis*, *Salix divaricata* and *Salix nankongensis* are on the ‘Red List of Endangered Species in China’ [20]. In situ conservation of *Populus nigra* has been conducted in Europe as part of the European Forest Genetic Resource Programme, over a huge land area and at considerable cost [21]. Whilst seed banking at −20 °C is the preferred means of ex situ conservation of plant species in general, the reputed short lifespan of Salicaceae seeds renders their *ex situ* conservation challenging.

Seed longevity is related to parental environment and seed morphology. Endospermic seeds of species from damp and cooler regions tend to have relatively shorter life-spans compared with those from warm arid regions [4,22]. However, far less is known about the lifespan variance in non-endospermic and short-lived seeds. Salicaceae species are distributed throughout the Northern Hemisphere [23] and widely grown in Europe, Scandinavia, the United States and Asia. There are 620 Salicaceae species worldwide, and 56% (i.e., 347) are present in China [24]. The wide geographical range of wild Salicaceae provides an excellent system for unravelling the genetic bases of environmental adaption [25,26] and seed longevity. Orthodox seeds gain their longevity gradually during the latter stages of development, increasing 30- to 50-fold, depending on the species and environment, as maturation progresses [27]. At the molecular level, key biochemical changes, such as the degradation of photosynthetic pigments, and the accumulation of heat shock proteins and late embryogenesis abundant (LEA) proteins, and soluble sugars (sucrose, raffinose, galactose, etc.) influence seed longevity.

Chlorophyll retention is suggested to be detrimental to longevity. Thus, in the model species Arabidopsis, *NON-YELLOW COLORING1* (*NYC1*), which acts at the first steps of chlorophyll breakdown, is strongly linked with seed longevity [28]. Moreover, *HSP* and s*HSP* genes, which accumulate during the late maturation stage, are regulated by transcriptional regulators such as *heat shock factors* (*HSFs*) upon stress [29]. Also, late embryogenesis LEA protein accumulation is another hall mark of seed maturation, such that the attainment of the longest lifespan at the final stage of seed maturation is co-incident with the accumulation of most LEA polypeptides [27]. Genes that regulate the acquisition of seed longevity are also highly enriched in defense-related processes based on their GO categories [30]. *WRKY* family genes, in particular, play an important role in regulating biotic defense. *WRKY3* is a positive regulator of defense against necrotrophic pathogen and longevity [30,31]. *WRKY33* has also been reported as a regulator of the jasmonate-related defense pathway [30]. Furthermore, seed longevity relies on antioxidants, such as tocopherols and glutathione, and secondary metabolites such as flavonoids [32]. Changes in the seed glutathione redox state coincide with the onset of ageing-induced seed viability loss in *Pisum sativum* [8].

To address the fundamental question of what environmental and molecular factors affect seed longevity in dry glassy states, we first stored seeds of Salicaceae species under dry and cool condition (dry room; 15 °C, 15% RH) versus ambient conditions to determine the seed longevity phenotype based on half-lives (P_50_). By storing Salicaceae seeds at −20 °C or in liquid nitrogen (LN) for 3 years after pre-equilibration to three RH levels (at 20 °C), we determined whether the seeds are short lived. Moreover, the variances in dry seed longevity among species were co-correlated with climate factors. Finally, transcriptome analysis was carried out to enable comparison during the ageing process of two *Populus* species, and a model of the relationship between climate, transcript profile and P_50_s kinetics was provided.

## 2. Results

### 2.1. Germination Decreased with Seed Dispersal

**Figure 1:**
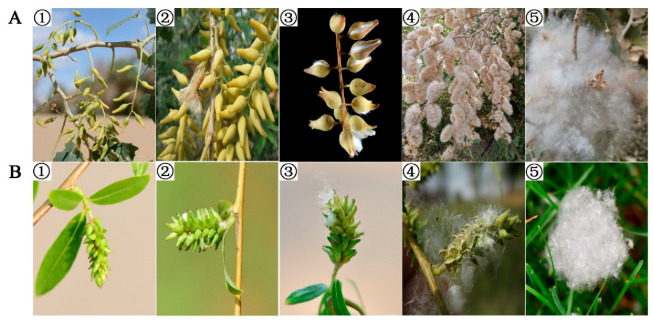
Maturation stages of *Populus euphratica* Oliv. (S1) (**A**) and *Salix babylonica* L. (S1) (**B**). Stages of capsule development were green capsules ①, just before dehiscence (yellowish capsules) ②, start of dehiscence ③, fully dehiscing ④ and dispersed ⑤.

As seed longevity is directly affected by the initial seed quality, which in turn is a function of the developmental stage, we first used four species to examine the optimize seed harvest time. Accordingly, we characterized the capsule development stage for four species (two of each genus) and evaluated seed viability and desiccation tolerance at five stages of capsules development: (1) green capsules; (2) just before dehiscence; (3) at the beginning of dehiscence; (4) fully dehiscent; and (5) at dispersal (Figure 1). At the green capsule stage, there was a significant decrease in viability on seed drying in *Salix psilostigma* (from 98% to 7%), *Populus euphratica* (S1) (from 85% to 42%) and *Populus davidiana* (from 86% to 67%). Only *Salix babylonica* (S1) seeds tolerated drying at this stage of capsule development. In contrast, 100% viability was recorded for all four species’ seeds before and after desiccation when seeds were collected from yellowish capsules, i.e., just before dehiscence (Table 1). At the start of capsule dehiscence (stage 3), high viabilities (>95%) post-desiccation were also obtained for seeds of all four species. However, a significant decrease in viability occurred at full dehiscence in *S. babylonica* (S1) (60%). After natural dispersal, the seeds of *P. davidiana* had 77% germination while germinability of *P. euphratica* (S1) and *S. babylonica* (S1) seeds had already decreased to c. 2% and %, respectively.

These data suggested that the optimal collecting time for Salicaceae species is just before the capsules dehisce and this procedure was followed for all harvested seed lots.

### 2.2. Decreased Storage Temperature and Humidity Increased Seed Longevity in Seven Salix Species

**Figure 2:**
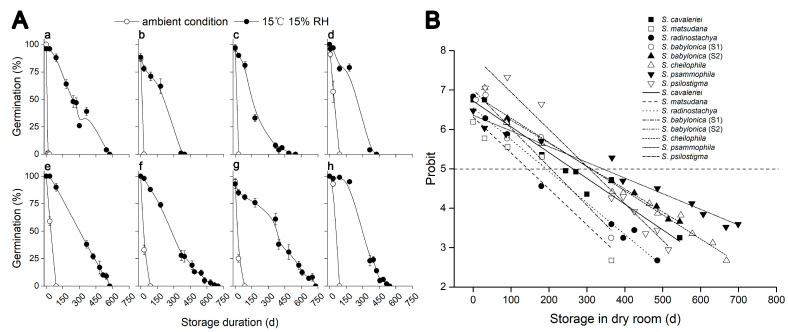
Total germination and relationship between storage time and germination of *Salix* species. (**A**) Germination after stored at ambient condition (open circle) or 15 °C 15% RH condition (solid circle). (**a**) *Salix cavaleriei*, (**b**) *Salix matsudana*, (**c**) *Salix radinostachya*, (**d**) *Salix babylonica* (S1) (**e**) *Salix babylonica* (S2), (**f**) *Salix cheilophila*, (**g**) *Salix psammophila*; (**h**) *Salix psilostigma*. Values are means of five replicates of 20 seeds ± SE. (**B**) Plot of the relationship between storage time (dry room storage at 15 °C, 15% RH) and germination (probit scale) for *Salix* species. *S. cavaleriei* (◼), *S. matsudana* (◻), *S. radinostachya* (●), *S. babylonica* (S1,◯), *S. babylonica* (S2,▲), *S. cheilophila* (△), *S*. *psammophila* (▼); *S*. *psilostigma* (▽).

To assess whether decreased temperature and humidity could extend *Salix* seed life span, we examined seven *Salix* species’ seed viability under two storage conditions. Germination of fresh seeds (0 d) was high in all species (>85%). Storage at both room temperature (RT) and in a dry room (15% RH, 15 °C) led to a decline in germination in all *Salix* species (Figure 2). However, dry room storage consistently showed greater seed longevity than storage at RT. By 14 d of storage at RT, germination of *Salix cavaleriei* had fallen to 1%, indicating a short life-span phenotype (Figure 2A(a)). Seeds of *S. babylonica* (S1, S2) (Figure 2A(d,e)) and *Salix psilostigma* (Figure 2A(h)) showed relatively good seed longevity, with 57%, 59% and 93%, respectively, after storage at RT for 30 d. Seeds of all seven *Salix* species lost viability in 90 d at RT.

When seeds of all species were stored in a dry room for 90 d, gemination remained above 70% (Figure 2A). A co-plot of germination (probit) against storage time revealed that when germination was 5 probits (i.e., 50% germination; P_50_), the interpolated seed longevities were 263, 144, 192, 205, 312, 309, 341 and 306 d for *S. cavaleriei*, *Salix matsudana*, *Salix radinostachya*, *S. babylonica* (S1), *S. babylonica* (S2), *Salix cheilophila*, *Salix psammophila* and *Salix psilostigma* seeds, respectively (Figure 2B).

These data indicated clearly that lowering the storage temperature and seed moisture (at equilibrium with 15% RH) improved seed longevity in all seven *Salix* species.

### 2.3. Decreased Storage Temperature and Humidity Increased Seed Longevity in Six Populus Species

**Figure 3:**
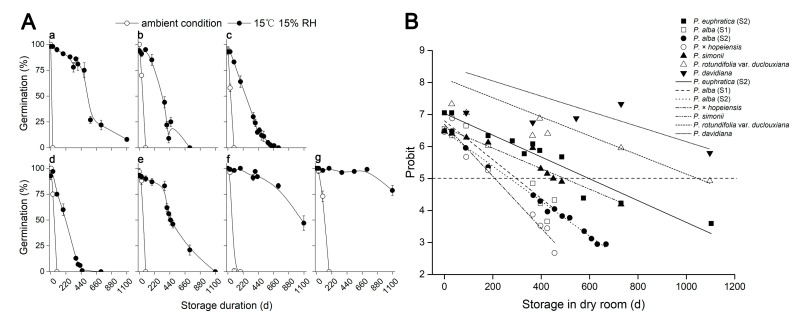
Total germination and relationship between storage time and germination of *Populus* species. (**A**) Germination after storage at ambient condition (open circle) or in a dry room at 15 °C 15% RH (solid circle). (**a**) *Populus euphratica* (S2), (**b**) *Populus alba* (S1), (**c**) *Populus alba* (S2), (**d**) *Populus × hopeiensis*, (**e**) *Populus simonii*, (**f**) *Populus rotundifolia* var. *duclouxiana*; (**g**) *Populus davidiana*. Values are means of five replicates of 20 seeds ± SE. (**B**) Plot of the relationship between storage time (stored at 15 °C, 15% RH condition) and germination (probit scale) for *Populus* species. *Populus euphratica* (◼), *Populus alba* (S1, ◻), *Populus alba* (S2, ●), *Populus × hopeiensis* (◯), *Populus simonii* (▲), *Populus rotundifolia* (△); *Populus davidiana* (▼).

To test whether decreased storage temperature and humidity increased longevity in *Populus* species, we examined six *Populus* species’ seed viability under two storage conditions. Control (0 d) seed germination was high (>95%) in all species. For *P. euphratica* (S2) seed, viability was totally lost by 30 d storage at RT; however, germination remained high (86%) after 1 year’s storage under the dry room condition (Figure 3A(a)). Similarly, seed viability in most species was lost within 3 months when stored at RT, except for *Populus davidiana* seeds which had 73% germination, indicating that the seeds are relatively long-lived. However, even seeds of this species totally lost viability after half a year (Figure 3A(g)).

A co-plot of germination (probit) against storage time in the dry room revealed that when germination was 5 probit (i.e., 50% germination), the interpolated storage half-lives (P_50_) were 597, 297, 271, 209, 497, 1047 and 1481 d for *P. euphratica* (S2), *Populus alba* (S1), *P. alba* (S2), *Populus × hopeiensis*, *Populus simonii*, *Populus rotundifolia* and *P. davidiana* seeds, respectively (Figure 3B).

These data showed that lower temperature and moisture content during storage of seeds improved seed longevity of all six *Populus* species and that dry seed life span in *Populus* tends to be greater than in *Salix* species.

### 2.4. Salix and Populus Seeds Maintain High Viability After Three Years Storage at Both −20 °C and in LN

**Figure 4:**
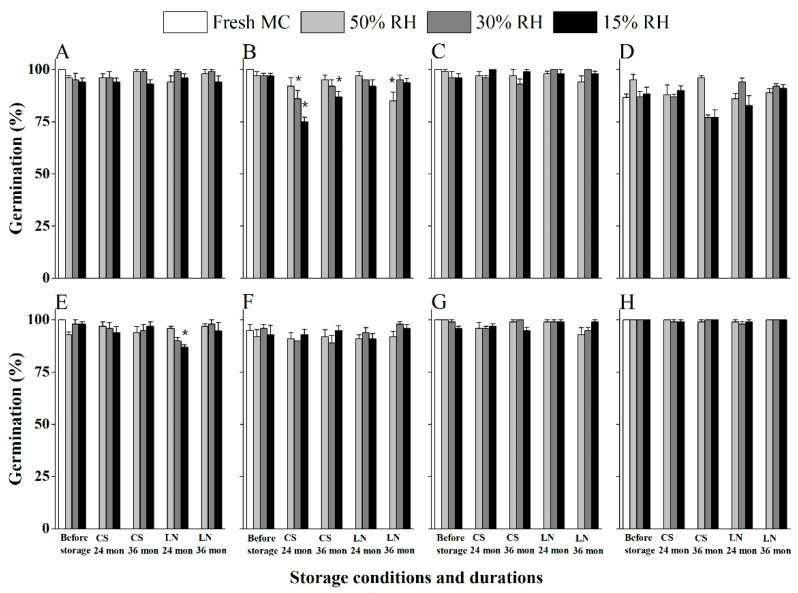
Germination of *Salix* species after 2 or 3 years of storage under cold (−20 °C) and ultra-cold liquid nitrogen (LN) conditions. (**A**) *Salix babylonica* (S1), (**B**) *Salix radinostachya*, (**C**) *Salix cheilophila*, (**D**) *Salix matsudana*, (**E**) *Salix wilhelmsiana*, (**F**) *Salix psammophila*, (**G**) *Salix cavaleriei* and (**H**) *Salix psilostigma.* Values are means of five replicates of 20 seeds ± SE and * denotes significant (*p* < 0.05) decreases in germination (viability) compared to the fresh seeds before storage, for each species.

**Figure 5:**
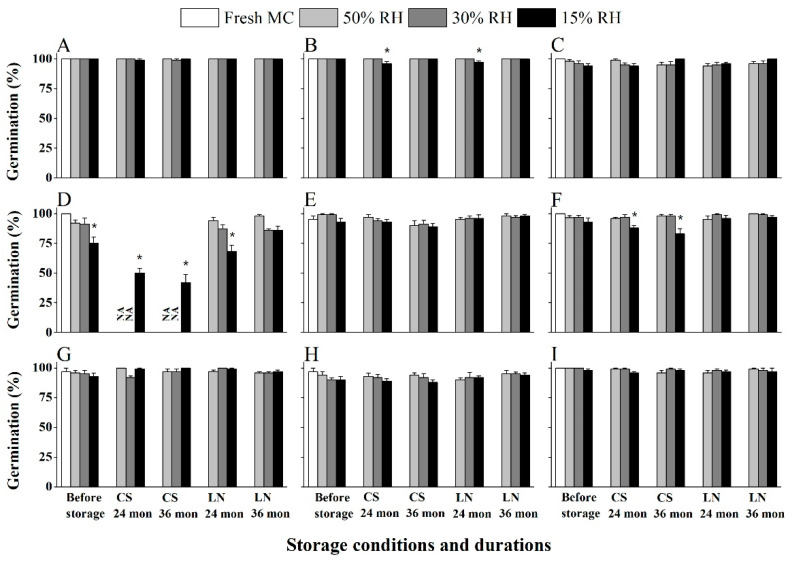
Germination before storage and after 2 or 3 years of storage under cold (−20 °C) and ultra-cold liquid nitrogen (LN) conditions. (**A**) *Populus davidiana*, (**B**) *Populus rotundifolia*, (**C**) *Populus alba* (S1), (**D**) *Populus tomentosa*, (**E**) *Populus alba* (S2), (**F**) *Populus × hopeiensis*, (**G**) *Populus simonii*, (**H**) *Populus cathayana*; (**I**) *Populus euphratica* (S2). Values are means of five replicates of 20 seeds ± SE and the * denotes significant decreases in germination (viability) compared to the fresh seeds before storage, for each species. NA signifies that data were not available for four samples.

Storage at RT and in the dry room suggested that seeds of all *Salix* and *Populus* species studied are relatively short-lived. Next, we explored whether seed longevity was also short under seed bank (−20 °C) and cryo bank (LN, −196 °C) conditions, using seeds equilibrated to three pre-storage RHs. Generally, lowering temperature from −20 °C to −196 °C increased seed longevity (Figure 4 and Figure 5). Fresh seeds in all species had > 85% germination. Seed germination decreased from 100% to 92% and 91% in *P. tomentosa* (Figure 5D) after drying to 50% and 30% RH, respectively, and significantly down to 75% when drying to 15% RH. There was no significant decrease in final germination after 3 years’ storage at both cold temperatures for 13 species (*S. babylonica* [S1], *S. cheilophila*, *S. matsudana*, *S. psammophila*, *S. wilhelmsiana*, *S. cavaleriei.*, *S. psilostigma*, *P. davidiana*, *P. alba*, *P. rotundifolia*, *P. simonii*, *P. cathayana*, *P. euphratica* [S2]). In contrast, significantly decreased seed germination levels were observed after 3 years’ storage at 15% RH and at −20 °C in three species: *S. radinostachya* (Figure 4B)*, P. × hopeiensis* (Figure 5F) *and P. tomentosa* (Figure 5D).

Overall, the data indicated that LN provided better long-term seed storage for Salicaceae species, and that pre-drying to 15% RH was potentially sub-optimal for some species.

### 2.5. Correlation Studies Between Seed Longevity P_50_ and Meteorological Data

**Figure 6:**
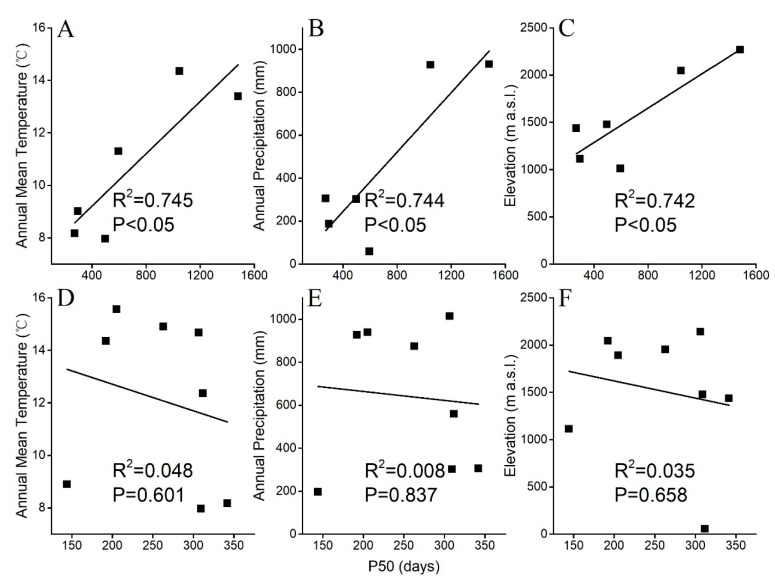
Correlation between seed longevity P_50_ as well as environmental conditions of the collecting sites of six *Populus* seedlots and eight *Salix* seedlots. Relationship between P_50_ and (**A**,**D**) annual temperature (°C), (**B**,**E**) annual precipitation (mm) and (**C**,**F**) elevation (m) in *Populus* (**A**–**C**) and *Salix* (**D**–**F**).

The conditions of the maternal environmental (such as annual temperature, annual precipitation and elevation) were tested as co-correlations of seed longevity P_50_. We selected the 14 locations from which the Salicaceae species were harvested to account for a broad range of environmental envelope in which the species grow, spanning altitudes from <150 to >2200 m a.s.l. (Table 2 and Table 3), and in which the seeds mature on the parent tree. We used the seed ageing curves at 15% RH and 15 °C (Figure 2 and Figure 3), generated over up to 3 years of storage. In *Populus* species, the P_50_ measure of seed longevity showed a strong positive correlation with the annual temperature (R^2^ = 0.745) towards 14 °C, precipitation (R^2^ = 0.744) towards 900 mm, and elevation (R^2^ = 0.742) towards 2200 m a.s.l. (Figure 6A–C). Thus, species from warm and wet regions produced relatively longer-lived seeds compared to congeners from colder and drier regions. However, seed longevity in *Salix* species showed no such correlations with average temperature (R^2^ = 0.048), precipitation (R^2^ = 0.008) and elevation (R^2^ = 0.035) (Figure 6D–F).

### 2.6. Transcriptome Analysis of P. euphratica (S2) (Shorter-Lived) and P. davidiana (Longer-Lived)

**Figure 7:**
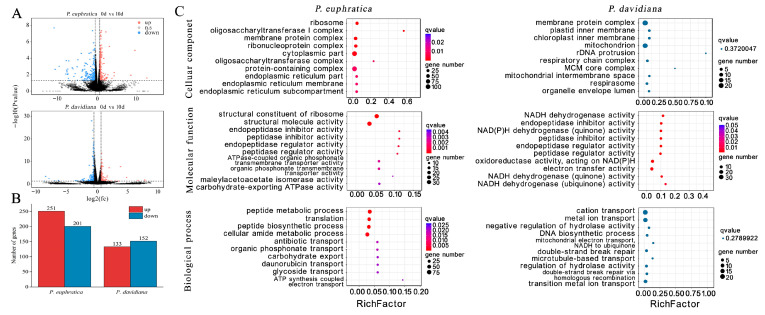
Differentially expressed genes (DEGs: FDR < 0.05 and absolute log 2 fold change ≥ 0.6) were identified for two different comparisons and GO enrichment analysis during ageing of *P. euphratica* and *P. davidiana* seeds. (**A**) Volcano plots depicting DECs within seed lots of *P. euphratica* (S2) and *P. davidiana* exposed to ageing processes. Red dots indicate significantly up-regulated genes, blue dots indicate significantly down-regulated genes, and black dots represent non-DEGs. (**B**) Up (red) and down (blue) DEGs for each comparison. (**C**) Enrichment results for the cellular component, molecular function and biological process category in the GO enrichment analysis. Seeds were aged for 10 d at 20 °C and 50% RH.

Because there was at least a two-fold difference in longevity between *P. euphratica* (S2) and *P. davidiana* under both ambient and dry room conditions (Figure 3), we then explored the molecular factors involved in this variation at an early stage of ‘dry’ seed ageing at 20 °C and 50% RH. For two species, we compared the gene expression in fresh seeds with that of seeds aged for 10 d (Figure 7). We used “FDR < 0.05 and |log2FC| ≥ 0.6” as the criteria for significant difference in gene expression (DEGs). During the ageing process of *P. euphratica* (S2; relatively shorter-lived), 456 DEGs were identified, of which 251 were up-regulated and 201 down-regulated (Data S1, Figure 7). In contrast, only 285 DEGs were identified when *P. davidiana* (relatively longer-lived) was compared before and after the ageing process, with 133 up-regulated genes and 152 down-regulated genes (Data S2, Figure 7). These results suggest that large transcriptomic differences existed between the two different species during ageing. The ageing process triggered considerable changes in gene expression in *P. euphratica* (S2), while only slightly affecting *P. davidiana* seeds.

Enrichment analysis highlighted significant involvement of cellular component (ribosome, oligosaccharyl transferase I complex, membrane protein complex, etc.), molecular function (structural constituent of ribosome, structural molecule activity, etc.) and biological processes (peptide metabolic process, translation, etc.) during ageing processes in *P. euphratica* seeds. On the other hand, only molecular functions, including NADH dehydrogenase activity, endopeptidase inhibitor activity, etc., were significantly involved in seed ageing processes of *P. davidiana* (Figure 7C, Data S3).

### 2.7. Late Maturation Genes Showed Dramatic Differences Between Species with Shorter- and Longer-Lived Seeds

To explore the genes involved in regulating the seed ageing process between shorter-and longer lived *Populus* species seeds, we first analysed the expression of transcription factors (TFs) that play critical roles in the regulation of this trait. Focusing on *P. euphratica* (S2) (shorter-lived seeds) during ageing, there were nine genes encoding TFs for which gene expression was significantly changed (Data S4). In contrast, only 16 unigenes encoding TFs showed significant changes following the ageing process of *P. davidiana*. The *WRKYs* families had the highest number of DEGs between 0 d and 10 d of ageing for *P. euphratica* (S2) seeds.

**Figure 8:**
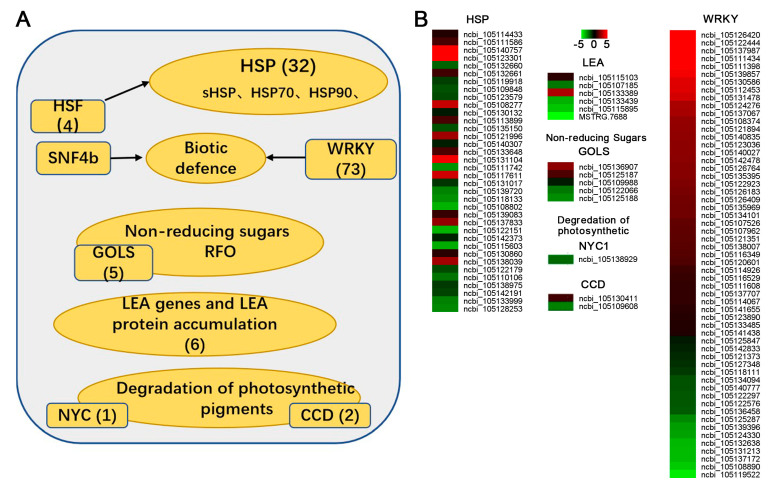
Differentially expressed late seed maturation related unigenes and their relative expression in shorter-lived (*Populus euphratica* [S2]) vs. longer-lived (*Populus davidiana*) seeds. (**A**) Putative unigenes involved in late seed maturation. The value in brackets indicates the number of unigenes annotated. (**B**) The heatmap shows the relative transcript level of each late seed maturation related unigene in *P. euphratica* (S2) vs. *P. davidiana*. The deferentially expressed unigenes (FDR < 0.05 and absolute log2 fold change ≥ 0.6) of the two seed lots before ageing.

In order to analyze the role of seed late maturation genes in ageing, we compared our results on *P. euphratica* and *P. davidiana* with that of another orthodox dicot species [27]. We identified 107 differential expressed genes (DEGs) when comparing seeds of the two species before ageing (Figure 8, Data S5). These included genes encoding *HSPs* (36), *late embryogenesis abundant (LEA) proteins* (6) and *WRKY* (57), as well as genes involved in the degradation of photosynthetic pigments (3) and non-reducing sugars (5). Gene expression of *HSP17.4*, *galactinol synthase 2-like*, *LEA5, LEA D29*, *WRKY 75* and *NYC1* has been validated using real-time PCR. The gene expression trend in both 0 d and 10 d aged seeds of *P. euphratica* (S2) and *P. davidiana* were in accordance with transcriptome results (Figure S1).

Regarding *HSP* families, four DEGs encoded for *heat shock factor proteins*, twenty for *small heat shock proteins* (*sHSPs*), eight for *HSP70* family proteins, two for *HSP80* family proteins and two for *HSP90* proteins. There were 15 *HSP* unigenes significantly up regulated in *P. euphratica* (shorter-lived) seeds compared to *P. davidiana* (longer-lived), with 21 *HSP* unigenes significantly down-regulated. Among six *LEA* related unigenes, four of them showed higher expression in *P. davidiana* (longer-lived) seeds. As for soluble sugars, three out of five DEG encoding *galactinol synthase* showed higher expression in *P. davidiana* (longer-lived) seeds before ageing (Figure 8). The degradation of photosynthetic pigments including chlorophyll b reductase (*NYC1*) and one carotenoid cleavage dioxygenase showed significantly higher expression in *P. euphratica* (shorter-lived) seeds.

In addition, the present study identified 57 differentially expressed *WRKY* unigenes involved in biotic defense. Two thirds of those DEGs showed higher expression in *P. euphratica* (shorter-lived seeds), while one third showed lower expression in *P. davidiana* (longer-lived) seeds.

The comparative data above indicated that gene expression characteristics associated with late seed maturation of *P. davidiana* (longer-lived) and *P. euphratica* (shorter-lived) were different before ageing. These findings strongly imply that seeds enter the glassy state already ‘pre-conditioned’ for longer or shorter lifespan.

### 2.8. Longer-Lived Seeds (Populus davidiana) Show Stable Levels of Glutathione Metabolism Genes During Ageing

**Figure 9:**
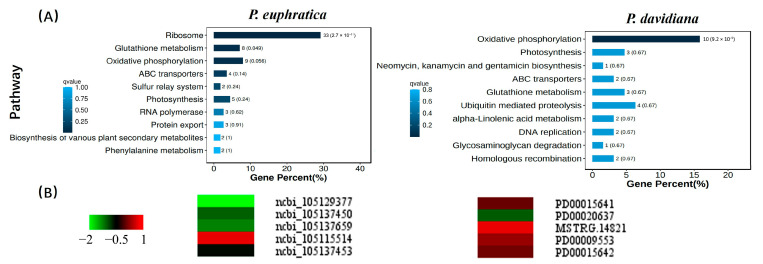
KEGG pathway and analysis of glutathione metabolism genes that were differentially expressed (DEGs: FDR < 0.05 and absolute log2 fold change ≥ 0.6) in shorter-lived (*Populus euphratica* [S2]) seeds following ageing. (**A**) KEGG enrichment pathway analysis. The dark blue indicates the enrichment results were significant (q value < 0.05). (**B**) Expression levels of glutathione metabolism genes that were significantly up,down-regulated in *P. euphratica* (S2) seeds following ageing (10 d vs. 0 d).

DEGs of *P. euphratica* (S2) and *P. davidiana* were analyzed for enrichment of KEGG terms (Figure 9, Data S6) to further understand the types and function of genes involved in ageing processes. KEGG enrichment analysis confirmed the significantce of rubosome, glutathione metabolism, oxidative phosphorylation, ABC transprots, sulfur relay system and photosynthesis when *P. euphratica* (shorter-lived) seeds were aged. In contrast, only photosynthesis as a biological process significantly enriched the longer-lived *P. davidiana* seeds (Figure 9A). Since glutathione metabolism is closely related to ROS, we compared in detail the homologue gene expression in *P. davidiana* and *P. euphratica* for fresh and 10 day-aged seeds (Figure 9B). In *P. euphratica* (shorter-lived) seeds, only one gene was significantly up-regulated and the rest were down-regulated with ageing. However, none of these genes showed significant changes following ageing in *P. davidiana* (longer-lived) seeds. These results suggest that longer-lived seeds (*P. davidiana*) have stable glutathione metabolism genes during the ageing process.

## 3. Discussion

### 3.1. Initial Seed Quality and the Glassy State Directly Affects Conservation Outcomes

Although some Salicaceae species are considered to have short-lived seeds, or even seeds that are desiccation sensitive, our findings for seeds stored for 3 years at −20 °C or in LN reveal this is not the case for the vast majority of the 17 seed lots across 16 species (Figure 4 and Figure 5). Maximizing the initial seed quality enhances their subsequent cold storage potential [33], as evidenced for another group of reputedly short-lived seeds, orchids [34]. For Salicaceae seeds, we optimized the collection time (Figure 1 and Table 1) and developed an efficient and benign post-harvest handling method (Figures S2 and S3) to ensure high initial germination (i.e., 94% of seedlots had >95% germination), which contributed to their successful long-term storage (Figure 4 and Figure 5). When the initial quality of seeds is around 85% rather than close to 100%, seed life span can be compromised. *S*. *alba* seeds collected at the point of release from yellowish capsules retained 93% germination after storage in LN with 6.7% MC. *S*. *matsudana* seeds retained 97% germination when held at −70 °C for 30 months with 10.5% MC germination [35]. Similarly, high initial seed quality is important for the maximum benefit of cryostorage (LN) of *Salix* and *Populus* species [36]. In contrast, *Populus koreana* seeds with an initial germination of 87% had reduced viability (75%) after desiccation to 9% MC, falling to around 40% when seeds were stored at room temperature for one week at 9–12% MC [18]. Moreover, *S. alba* seeds with a low initial germination of 75% fell to c. 40% germination after only 150 d storage in −20 °C [35].

Over 90% of tested *Salix* species exhibited complete loss of viability within three months under ambient storage conditions (room temperature), with seed longevity ranging from mere days to several months (Figure 2 and Figure 3). Notably, controlled low-humidity (15% RH) and cool temperature (15 °C) conditions significantly enhanced seed preservation efficacy, indicating that the transfer of Salicaceae seed from room conditions to a conventional seed bank drying room is beneficial and should be implemented as soon as seed arrives at the seed bank.

Our analysis of 16 Salicaceae species revealed that seeds dried at 50% and 30% RH (c. 6–9% MC; Figure S3) retained high viability following 3 years of storage at −20 °C, which indicates an orthodox-type seed storage behavior (Figure 4 and Figure 5). This aligns with the National Tree Seed Center of Canada’s longitudinal study demonstrating sustained *Populus/Salix* seed viability under comparable −20 °C storage [37]. However, two species and one hybrid [*S*. *radinostachya* (Figure 4B), *P*. *tomentosa* (Figure 5D), and *P. × hopeiensis* (Figure 5F)] exhibited significant germination decline (*p* < 0.05) under 15% RH storage, highlighting moisture-dependent viability thresholds. Similar results were found in *S. alba*, which maintained high vigor when stored with 6.7% MC whilst germination decreased with further dehydration to 4.3% [35]. These results generally corroborate the established 4–10% MC window as being optimal for Salicaceae seed storage, and highlights a potential risk to viability loss of lower MC achieved with 15% RH conditions for some seed lots [37].

As for LN storage, for those seeds that are sensitive to lower MC (15% RH), such as *P. tomentosa* (Figure 5D), LN storage for 3 years improved survival (86% germination) compared to that at −20 °C (only 42%). It was shown for *P. nigra* seeds that 0.07–0.17 g H_2_O/g DW (equal to 6.5%–14.5% MC) was a safe MC range for overnight cryopreservation [21]. Similar results had also been reported for lower vigor seed lots of *S. alba* and *S. matsudana* (75% initial normal germination) when stored with 9% MC; immersion in LN resulted in no viability loss over 5 months, whilst germination decreased to 35–40% after storage at −20 °C for the same time [35]. Such sensitivity to conventional seed bank temperature storage suggests that some Salicaceae species seeds fit exceptionality factor 3 of ‘Exceptional species’ [38].

In summary, the optimal MC for *Populus* and *Salix* seeds for long-term storage appears to be between 6 and 9% (around 30% RH–50% RH), and seeds lots with high vigor could survive conventional seed banking for many years, but longevity should be improved by LN storage. Moreover, dry room conditions (15% RH/15 °C) not only improves seed lifespan but separates out species’ differences in longevity, enabling ageing mechanisms in the dry state to be explored in relatively short-lived seeds.

### 3.2. Transcriptome Analysis of Populus Dry-Stored Seeds Reveals the Importantce of Late Maturation Genes in Seed Lifespan

*Salix* seeds equilibrated to 60% RH at 20 °C have already entered the glassy state according to seed thermal profiling by DSC (Figure S4), and the position of the seeds in sorption zone II of the water sorption isotherm (i.e., no free water present; Figure S5). These conditions ensured that our dissection of seed longevity mechanisms was directly relevant to dry-stored seeds, unlike when seed viability loss is accelerated at high RH and temperature.

Transcriptional analysis revealed that considerable differences in gene expression existed between the relatively longer-lived seeds of *P. davidiana* and shorter-lived seeds of *P. euphratica*. These differences were particularly evident at the level of transcription factors, and genes expressed in late seed maturation of orthodox seeds.

In the present study, 57 *WRKY* genes showed significant differences between mature and high-quality seeds of the two *Populus* species (Figure 8). Two thirds of these genes were expressed more highly in the shorter-lived species (*P. euphratica*), and the other third had elevated expression in the longer-lived species (*P. davidiana*). These findings indicate that there is potentially a complex role for *WRKY* TF families in ageing processes. Considerable research has shown that *WRKY*s, and associated networks, have both positive and negative regulatory roles in various abiotic stresses [39]. *CiWRKY75–1* and *CiWRKY40–4* enhance drought tolerance and delays leaf senescence, respectively [40]. *AtWRKY6*, *AtWRKY53* and *OsWRKY45* positively regulate leaf senescence [41]. *SlWRKY39* and *PgWRKY65* were found to enhance dehydration and salinity stress responses in tomato and pearl millet [42]. *TaWRKY49* is suggested to have a negative regulation role in the seedling-plant resistance to Pst (HTSP) under high temperature [43]. Negative regulation of *CaWRKY40b* has also been reported in the response of pepper plants to the pathogenic bacterium *Ralstonia solanacearum* [44]. WRKY proteins also play an important positive or negative regulatory role as part of a precise regulatory network that controls seed dormancy and germination [45,46].

We observed a two-fold higher *NYC1* expression in seeds of the longer-lived species (*P. davidiana*) compared to the shorter-lived species *(P. euphratica*) (Figure 8). Degradation of the *NYC1* gene was reported to be responsible for the degradation of photosynthetic pigments, including chlorophyll and carotenoids, which is one of the most visible changes during seed maturation [27]. Our findings corroborate the observation that seeds of the *nyc1 nol* double mutants contain 10-fold more chlorophyll than the wild type have strongly reduced longevity [22].

*LEA1* and *LEA5* gene expression was significantly higher in longer lived pupolar species (*P. davidiana*) (Figure 8). *Late embryogenesis abundant* (*LEA*) *proteins* were reported to correlate with seed longevity [47]. The function of *LEA proteins* also varied during seed maturation. Certain *LEA proteins* could promote membrane fluidity and integrity [48]. Combined with sucrose and oligosaccharides, some *LEA proteins* could also participate in the glass formation during further dehydration of seed late maturation [49]. A two-fold reduction in *Arabidopsis thaliana* seed longevity was observed in the *LEA14* mutants with strong reduction in transcript abundance [47]. Four of the most abundant seed *LEA proteins* accumulated with seed longevity [50].

*HSP* and *sHSPs* accumulated during late seed maturation, although they are different in structure and size, but are common in terms of their chaperone activity. Chaperone function may therefore play a positive role in seed longevity [27]. Similary, we found 36 *HSP* genes had significantly different expression between longer-lived (*P. davidiana*) and shorter-lived (*P. euphratica*) seeds (Figure 8). *sHSP18.2* is an ageing responsive protein that has the ability to improve seed longevity by reducing deleterious ROS accumulation in seeds [51]. The other two chaperons *HSP70* and *HSP90* that assist in protein folding during abiotic stress responses are also thought to be involved in the seed longevity module of *Medicago truncatula* [52,53].

*HSF8* was significantly more highly expressed in long-lived seeds (*P. davidiana*) (Figure 8)*. Heat shock TF (HSF) proteins* are reportedly correlated with seed longevity. *HSFA8* plays a role in oxidative stress and ROS signaling under stress conditions, functioning as cytosolic peroxide sensors, such as H_2_O_2_, via oxidation of key Cys residues and formation of disulfide bonds [54]. During drought conditions, *HSFA8* promotes the accumulation of flavonoids to scavenge ROS and also interacts with *HSP90* to inhibit its binding activity and transcriptional activation [55].

DEGs during ageing processes were identified and subjected to GO/KEGG enrichment analysis. It was suggested that when seeds aged under dry (glassy) conditions, the formation of reactive oxygen species (ROS) would occur at a slow rate through auto-oxidative processes [1]. The accumulation of ROS would then result in protein, lipid and DNA damage in seeds. The present KEGG analysis revealed significant enrichment of the glutathione metabolism pathway, to the extent that it was the second most enriched in KEGG terms during ageing (Figure 9). In contrast, the genes involved in this pathway in long-lived seeds (*P. davidiana*) did not show any significant changes during the ageing process. Glutathione S transferases are known to be involved in both abiotic and biotic stresses [7,56]. For example, oxidative damage caused by drought, NaCl, and Cd stresses is enhanced in tobacco lines with overexpression of *PpGST* [57]. Also, when *GST* and *GPX* genes are overexpressed in *OsGSTL2* lines, there is reduced superoxide stress [58]. These results indicate that *P. euphratica* (shorter-lived) seeds may have already been exposed to abiotic stress at the early stage of ageing processes. Moreover, DEGs are also significantly involved in cellular and molecular functions and biological processes during ageing of *P. euphratica* seeds, potentially affecting structual molecular activity and membrane protein complexes. However, no DNA repair-related genes were found during ageing, which indicates that the glassy state limited the DNA repair process.

All the data above indicate that genes related to late maturation of seeds and oxidative stress might be the most important contributors to differences in seed longevity between *P. davidiana* and *P. euphratica*.

### 3.3. Seed Lifespan of Populus Species Was Strongly Correlated with Environmental Factors

Seed longevity is influenced by the maternal environment, i.e., the collection site, during the later stages of seed development. But the pattern of influence may vary with the scales of the sampling across environments. For example, when comparing seed P_50_s of six crop species collected at sites from Europe to Australia, the seeds that showed longer shelf lives in the seed bank (freezer) came from warm/drier (Australia) rather than cold/wet (Europe) conditions [6]. Similar results were obtained from a comparison of longevity under accelerated ageing conditions for seeds of 195 species collected from environments with large annual temperature (c. −10 °C to 42 °C) and annual rainfall (c. 0–3800 mm) variations [4]. In the latter study, endospermic seeds tended to be shorter-lived. In contrast, for *Populus* species (Figure 6) sampled across an annual temperature range of 8 °C to 14 °C, and annual rainfall of c. 60–930 mm, it was clear that these non-endospermic seeds tended to be long-lived in the dry storage when the maternal environments (seed collection sites) were warmer (above 14 °C) and wetter (above 800 mm) (Figure 6A,B). Positive correlation (R^2^ = 0.567) was also found between annual mean temperature and annual precipitation, and this correlation is a general climate phenomenon in China. Thus, we speculated that in east Asia, *Populus* species in cooler and drier areas tend to produce short lived seeds (Figure S6). However, in this study, seed longevity of *Salix* species that were sampled from overlapping environments showed no correlation either with annual mean temperature or annual precipitation (Figure 6D,E). Thus, the species and environment interaction for seed longevity may vary with genus, or is not evident in *Salix* as the range of sampling environments was narrower than that of *Populus*. Future studies should focus on sampling *Salix* seeds from species growing over wider elevation and environment ranges to enable a further dissection of the molecular basis of longevity of short-lived seeds in the dry glassy state.

**Figure 10:**
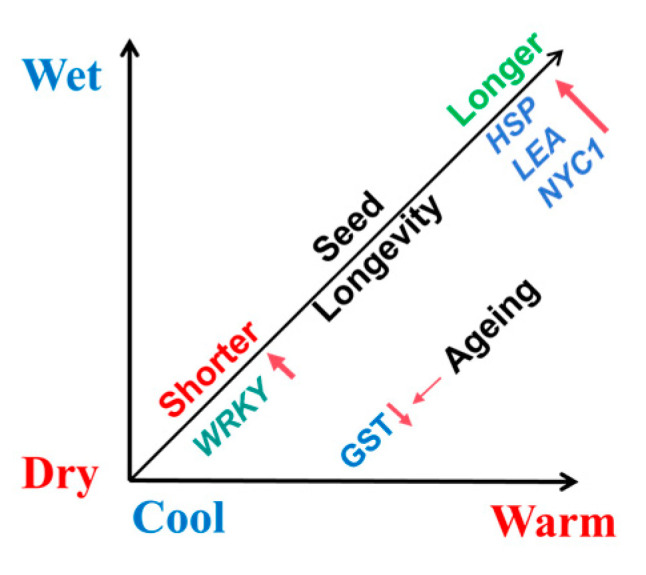
A graph of the relationship between climate, transcript profile and P_50_ kinetics of *P. euphratica* and *P. davidiana*. Up arrows: most genes high expression; Down arrows: gene expression decreased.

In a summary, *Populus* species from warm wet environments tended to produce longer-lived seeds in dry storage (Figure 10). We found that 60% of *HSP*, 67% of *LEA* genes and *NYC1* showed higher expression in *P. davidiana* seeds. While 70% of *WRKY* genes significantly higher expressed in *P. euphratica* seeds. The ageing process of dry glassy states caused downregulation of *glutathione metabolism* genes, which is related to oxidative stress in *P. euphratica* (shorter-lived) seeds, but not in longer-lived seeds after the same ageing times.

## 4. Materials and Methods

### 4.1. Capsule Collection and Seed Cleaning

Capsules at different maturity stages (green, just before dehiscence, slightly dehiscing, fully dehiscing, scattered) (Figure 1) were collected for two *Salix* species (*S*. *babylonica* L. [seed lot 1; S1] and *S*. *psilostigma* Anderss.) and two *Populus* species (*P*. *euphratica* Oliver [S1] and *P*. *davidiana* Dode). Also, capsules were collected just before dehiscing from eight *Salix* species and eight *Populus* species. All capsules of similar maturity from one species were pooled in a large zip lock bag. The bags were not compressed but contained air to avoid mildew growth, and transported within 3 d to the Germplasm Bank of Wild Species (Kunming, Yunnan Province).

On arrival, capsules were placed in a dry room operating at 15 ± 2 °C and 15 ± 2% relative humidity (RH) for 24 or 48 h to promote full opening. During this period, capsules were occasional turned and stirred to facilitate uniform drying and seed release. For three species (*P. euphratica* [S2], *S. babylonica* [S1], and *S*. *cavaleriei* H. Lév.), seed hairs were removed by three methods: hand stripping, rubbing and vacuum negative pressure [59]. Only the vacuum negative pressure method was used for the other species. This cleaning method involved passing seeds through different sieve sizes (0.18–0.25 mm and 2.36–3.35 mm mesh) under a negative air pressure of 15–30 kPa for 2–3 min.

An experimental design and workflow of the ageing process is shown in Figure S8.

### 4.2. Seed Desiccation Treatments, Storage Conditions and Germination Test

Seeds were dried for 2–3 d under ambient conditions (around 20 °C, 50% RH), or in sealed containers above LiCl solutions generating 30% or 50% RH at 15 ± 2 °C, or in a dry room operating at 15 °C, 15% RH. After equilibration was achieved for these RHs, seeds were stored under ambient conditions in a dry room (for up to 1100 d), at −20 °C or in LN (for up to 3 y). Seed RH was determined using a Rotronic HC2-AW probe attached to a Hygrolab C1 unit (Rotronic Ltd., Crawley, UK). Seeds that had been stored at −20 °C or cryopreserved in LN were rewarmed at room temperature for 30 min prior to germination testing (see below).

At each sampling time, 5 replicates of 20 seeds were sown on 1% agar–water medium in 90 mm diameter Petri dishes, and the dishes were incubated at 20 ± 1 °C over a 12 h per day photoperiod (illumination: 22.2 μmol m-2 s-1 generated by cool white fluorescent lamps). Germination, defined as radicle emergence of at least 2 mm, was scored every 7 d until at least 35 d. For all tests, no germination occurred during the final 7 d of this period and the test concluded at 35 d. Cut tests were performed on the non-germinated seeds and the vast majority were found to be soft and unviable. Seed germination was expressed as a percentage of the total number of seeds sown.

### 4.3. Water Sorption Isotherm Construction

Water sorption isotherms at 20 ± 2 °C were constructed for seeds of five species and one hybrid: *P. alba* (S1), *P.* × *hopeiensis*, *P*. *tomentosa* Carrière, *S. babylonica* (S2), *S. cavaleriei* H. Lév and *S. psilostigma*. For each species, 3 × 20 seeds were placed above LiCl solutions generating RHs between 15% and 100% (above water) for 3 d. The water activity (aw) was calculated as RH/100. After equilibration, the MCs (three replicates of 20 seeds) were determined gravimetrically by drying the seeds at 103 °C for 17 h (International Seed Testing Association, http://www.seedtest.org, accessed on 10 January 2025).

### 4.4. Meteorological Data of the Sampling Sites

Meteorological data including annual temperature and precipitation were obtained from the WorldClim database (www.worldclim.org) for each location.

### 4.5. Seed Ageing and RNA Extraction, Library Construction and Sequencing

Seeds of *P. euphratica* (S2) and *P. davidiana* were aged at 50% RH and 20 °C in an incubator and viability was assessed by germination testing as outliened above. Three biological replicates of 50 mg seeds were taken at two ageing stages (0 and 10 d), immediately frozen in LN and ground to a fine powder. Total RNA was extracted using the Trizol reagent kit (Invitrogen, Carlsbad, CA, USA) based on the provided instructions and as described earlier [60]. In brief, RNA quality was assessed on an Agilent 2100 Bioanalyzer (Agilent Technologies, Palo Alto, CA, USA) and checked using RNase-free agarose gel electrophoresis. After total RNA was extracted, mRNA was enriched by oligo (dT) beads. Then, the enriched mRNA was fragmented into small pieces using a fragmentation buffer and reverse transcribed to cDNA using the NEBNext Ultra RNA Library Prep Kit for Illumina (NEB7530, New EnglandBiolabs, Ipswich, MA, USA). The purified double-stranded cDNA fragments were end repaired, a base added, and ligated to Illumina sequencing adapters. The ligation reaction was purified with the AMPure XP Beads (1.0×) and polymerase chain reaction (PCR) amplified. The resulting cDNA library was sequenced using Illumina Novaseq6000 by Gene Denovo Biotechnology Co. (Guangzhou, China).

### 4.6. Differentially Expressed Genes (DEGs) and Pathway Enrichment Analysis

A FPKM (fragment per kb of transcript per million mapped reads) value was calculated to quantify gene expression abundance and variations, using RSEM software [61]. Differential expression of genes was performed by DESeq2 [62] software for two different contrasts (*P. euphratica* [S2] and *P. davidiana* seeds ageing for 0 day and for 10 d). Differentially expressed genes/transcripts (DEGs) were counted as genes/transcripts with a false discovery rate (FDR) below 0.05 and absolute log2 fold change ≥ 0.6. All DEGs were mapped to GO terms in the Gene Ontology database (http://www.geneontology.org/) for GO enrichment and pathway analysis. Gene numbers were calculated for every term, with significantly enriched GO terms in the DEG list (compared to the genome) defined by the hypergeometric test. The calculated *p*-values were subjected to FDR correction, taking FDR < 0.05 as a threshold.

### 4.7. Differential Scanning Calorimetry

The state of water and lipids in seeds of four species (*P. euphratica* [S2], *P. davidiana*, *S. matsudana*, *S. psammophila*) was determined by differential scanning calorimetry (DSC) after the seeds had been equilibrated at 50% RH and 20 °C for 3 d. A DSC (DSC2500 TA instruments, New Castle, DE, USA) was calibrated using indium. Three replicates of c. 4 mg of seed of each species were placed in pre-weighed aluminum pans, non-hermetically sealed but crimped with pre-weighed aluminum caps. Samples were warmed from room temperature to 60 °C and held (isothermally) at that temperature for 5 min, and then cooled at 10 °C min^−1^ to −80 °C, maintained isothermally for 5 min and then rewarmed at 10 °C min^−1^ to 60 °C. The melting peak temperatures, onset and endset temperatures, as well as enthalpies for lipid transitions in this dry material, were analyzed using the TA instruments TRIOS (V 4.4) software, in relation to the scanning baseline.

### 4.8. qPCR Test

Total RNA was extracted from the seeds of both fresh and aged seeds (10 d) of *P. euphratica* and *P. davidiana* using the FastPure Universal Plant Total RNA Isolation Kit-RC411 (Vazyme Biotech, Nanjing, China). The quality of total RNA was assessed with 1% agarose gel electrophoresis, and the concentration was determined using nano-volume spectrophotometry (Scan Drop, Jena, Germany). The cDNA was synthesized using TransScript^®^ All-in-One First-Strand cDNA Synthesis SuperMix for qPCR Kit (TransGen Biotech, Beijing, China). Quantitative real-time PCR (qRT-PCR) analysis was used to assess the target genes’ expression level in both fresh and aged seeds (10d) of *P. euphratica* and *P. davidiana*. Three technical and two biological replicates were performed for each gene. *ACTIN7* was employed as an internal reference gene for normalization of the threshold value (Ct) of the target genes due to its stable expression through the ageing process (Figure S7). Relative expression levels were calculated using the 2^−ΔΔCt^ method. The primers are listed in Table S1.

### 4.9. Data Analysis and Presentation

The germination data conforming to normal distribution and homogeneity of variance were tested using one-way ANOVA followed by pairwise comparisons of S-N-K (Student–Newman–Keuls) test. Differences at a level of *p* < 0.05 were considered to be significant. The germination percentage values were converted to probit data (Figure 2, Figure 3 and Figure 6). After plotting the satter data, we performed a linear fit using R (version R 4.2.2) language to interpoloate the lines. The correlation between probit data and meteorological data was analyzed using R (R: A Language and Environment for Statistical Computing, n.d., https://www.gnu.org/copyleft/gpl.html (accessed on 12 March 2024)). It was used for statistical analyses of the data.

## 5. Conclusions

In conclusion, seed longevity of short-lived willow and poplar was maximised by harvesting just before capsule dehiscence and using negative pressure to remove seed hairs. At 6–9% moisture content, most species retained viability after 3 years in storage at −20 °C or in liquid nitrogen. Dry room storage (15% RH, 15 °C) revealed interspecific seed lifespan differences. Notably, poplar species from warm/wet environment exhibited longer dry longevity than willow. Transcriptomes of *Populus davidiana* (longer-lived) and *P. euphratica* (shorter-lived) demonstrated late seed maturation genes (*HSPs*, *LEAs* and *WRKYs*) and oxidative stress-related genes as critical determinants of lifespan differences in the glassy state. These data will provide useful insights in understanding study ageing in the glassy state, contrasting with traditional accelerated ageing methods.

## 6. Patents

Patent ZL202211107382.9 was the result of the work reported in this manuscript.

## Figures and Tables

**Table 1:** The effect of collecting seeds at different maturity stages on seed germination. Germination was assessed on fresh seeds and on seeds that had been dried at equilibrium at 15 °C, 15% RH.

Species		Maturity Stages	Green Capsules	Yellow Capsules	Start of Dehiscence	Fully Dehiscing	Dispersed
	Water Status						
*Populus euphratica* Olivier (S1)	fresh seeds		85 ± 5.77 a	100 ± 0 a	98.33 ± 1.67 a	81.67 ± 8.33 a	1.67 ± 1.67 b
	dry seeds		42.5 ± 4.33 c *	100 ± 0 a	96.67 ± 1.67 a	78.33 ± 2.89 b	0 ± 0 d
*Populus davidiana* Dode	fresh seeds		86 ± 8.57 a	100 ± 0 a	99 ± 1 a	90 ± 2.24 a	77 ± 7 a
	dry seeds		67 ± 4.64 c	100 ± 0 a	100 ± 0 a	86 ± 1.87 b	71 ± 3.67 c
*Salix babylonica* L. (S1)	fresh seeds		80 ± 5.70 a	100 ± 0 a	98.67 ± 1.33 a	60 ± 7.64 b	26.67 ± 5.70 c
	dry seeds		86 ± 4.58 b	100 ± 0 a	95.33 ± 0.67 a, b	60 ± 2.89 c	18 ± 1.15 d
*Salix psilostigma* Anderss.	fresh seeds		98.33 ± 1.67 a	100 ± 0 a	100 ± 0 a	96.67 ± 1.67 a	NA
	dry seeds		7 ± 2.54 c *	100 ± 0 a	100 ± 0 a	90 ± 2.74 b	NA

Values are mean ± SE for five replicates of 20 seeds. Values in the same row with a different letter are significantly different (*p* < 0.05). NA, not available. * denotes significant decreases in germination compared to the fresh seeds before storage, for each species at different developmental stages.

**Table 2:** Geographical details of ten seed lots of *Populus* studied across seven species and one hybrid species.

Species	Collection Date (y/m/d)	Longitude	Altitude (m)	1000 Seeds Weight (mg)
*Populus alba* L. (S1)	2020/04/23	38°25′ N; 106°10′ E	1077	379.6
*Populus alba* L. (S2)	2020/04/13	37°44′ N; 107°19′ E	1378	312.0
*Populus euphratica* Oliver (S1)	2019/09/20	41°9′ N; 86°6′ E	820	NA
*Populus euphratica* Oliver (S2)	2020/09/18	40°32′ N; 81°17′ E	960	61.2
*Populus × hopeiensis* Hu & H.F. Chow	2020/04/13	37°44′ N; 107°19′ E	1377	197.2
*Populus simonii* Carr.	2020/04/15	37°44′ N; 107°04′ E	1467	509.6
*Populus davidiana* Dode	2020/03/29	25°14′ N; 102°44′ E	1915	110.0
*Populus rotundifolia* Griff	2020/05/15	25°08′ N; 102°44′ E	1882	136.4
*Populus tomentosa* Carrière	2020/04/22	38°28′ N; 106°12′ E	1105	311.2
*Populus cathayana* Rehd.	2020/07/13	41°22′ N; 111°44′ E	619	581.2

NA, not available. S, seed lot.

**Table 3:** Geographical details of nine seed lots of *Salix* studied across eight species.

Species	Collection Date (y/m/d)	Longitude	Altitude (m)	1000 Seeds Weight (mg)
*Salix babylonica* L. (S1)	2020/03/29	25°01′ N; 102°40′ E	1856	131.2
*Salix babylonica* L. (S2)	2020/04/19	40°00′ N; 116°19′ E	35	132.0
*Salix cavaleriei* H. Lév	2020/09/25	25°04′ N; 102°22′ E	1768	219.6
*Salix matsudana* Koidz.	2020/04/	38°30′ N; 106°15′ E	1104	100.8
*Salix radinostachya* C.K. Schneid.	2020/05/25	25°08′ N; 102°44′ E	1931	68.8
*Salix cheilophila* C.K. Schneid.	2020/04/15	37°44′ N; 107°04′ E	1468	76.4
*Salix psammophila* C. Wang & Chang Y. Yang	2020/04/13	37°44′ N; 107°19′ E	1396	76.4
*Salix wilhelmsiana* M. Bieb.	2020/05/11	38°25′ N; 106°10′ E	1115	62.0
*Salix psilostigma* Andersson	2020/05/23	25°13′ N; 99°17′ E	2299	96.8

S, seed lot.

## Data Availability

All data available are within the article and Supplementary Information. Sequence data used in this article can be found on the National Center for Biotechnology Information website (www.ncbi.nlm.nih.gov) under the following accession project number: PRJNA1313092.

## Supplementary Materials

The following supporting information can be downloaded at https://www.mdpi.com/article/10.3390/plants14182861/s1: Figure S1: Real-time PCR validation of *HSP17.4*, *Galactinol synthase 2-like*, *LEA5*, *LEA D29*, *WRKY 75* and *NYC1* during ageing process of *P. euphratica* (S2) and *P. davidiana* seeds. Figure S2: Negative pressure method to clean seeds; Figure S3: Effect of cleaning method on germination of *populus euphratica, salix babylonica* and *salic cavalerei* seeds; Figure S4: DSC warming (A and C) and cooling (B and D) thermograms showing lipid phase changes in two *Salix* species seeds (A and B) and two *Populus* species seeds (C and D); Figure S5: Relationship between equilibrium moisture content (MC) and water activity (a_w_) at 15 ± 2 °C; Figure S6: Correlation between annual temperature as well as annual precipitation of the collecting sites of six *Populus* seedlots. Figure S7: Effect of 10 d ageing on germination of *P. euphratica* (S2) and *P. davidiana* seeds. Figure S8: Experimental design and workflow of the seed ageing; Figure S9: Amplification plot of seven genes during ageing process of *P. euphratica* and *P. davidiana*; Figure S10: Melting curve of seven genes during ageing process of *P. euphratica* and *P. davidiana*; Table S1: Primers used for qRT-PCR in this research; Table S2: Quality control of raw reads of *Populus euphratica*; Table S3: Quality control of raw reads of *Populus davidiana*; Data S1: *P. euphratica* 0 d vs. 10 d; Data S2: *P. Davidiana* 0 d vs. 10 d; Data S3: GO enrichment; Data S4: TF genes; Data S5: Late maturation gene expression; Data S6: KEGG pathway genes; Data S7: Glutathione metabolism-related genes.
