# Supplementary material for: Environmental Factors, Developmental Genes and Oxidative Stress Determine Inter-Species Variability in Seed Longevity in Salicaceae
Source: Plants (Basel). 2025 Sep 13;14(18):2861. doi: 10.3390/plants14182861 (PMC12473942; doi:10.3390/plants14182861)
Supplement: Supplementary file 1 [file plants-14-02861-s001.zip › Chen et al 2025 Final Plants Supplemental Figures and tables.pdf]

Supplemental Figures and Tables

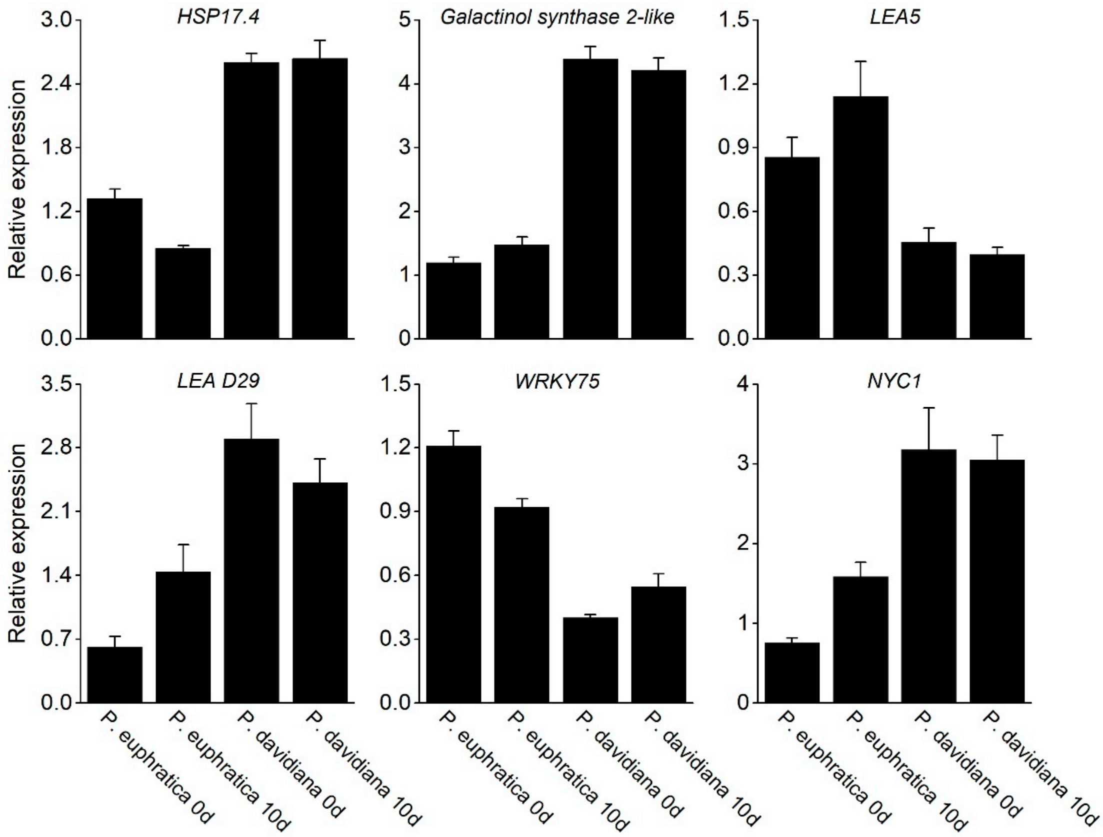

Figure S1. Real time PCR validation of *HSP17.4, galactinol synthase 2-like, LEA5, LEA D29, WRKY 75* and *NYC1* during the aging process of *P. euphratica* (S2) and *P. davidiana* seeds. Actin was used as the reference gene. Ageing samples were held at 20 ℃, 50 % RH for 10 d.

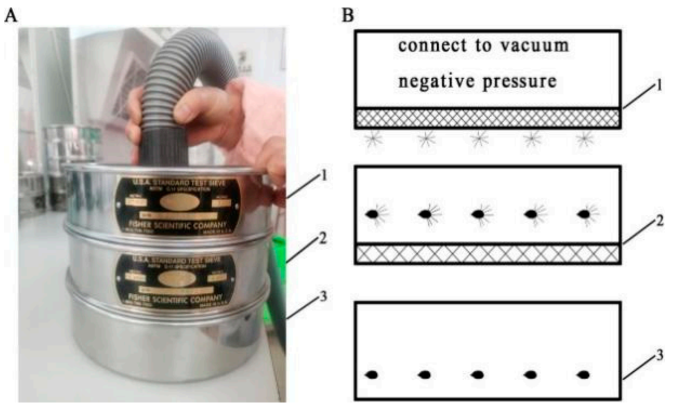

Figure S2. Negative pressure method of seed cleaning to remove the seed hairs, i.e. cotton. (A) General set up of two sieves and collecting container (A), and workflow of seeds during cleaning (B). Seeds with cotton attached were placed in the middle sieve (2) and the vacuum applied. The cotton is trapped by the upper sieve (1) as the seeds collect in the bottom container (3).

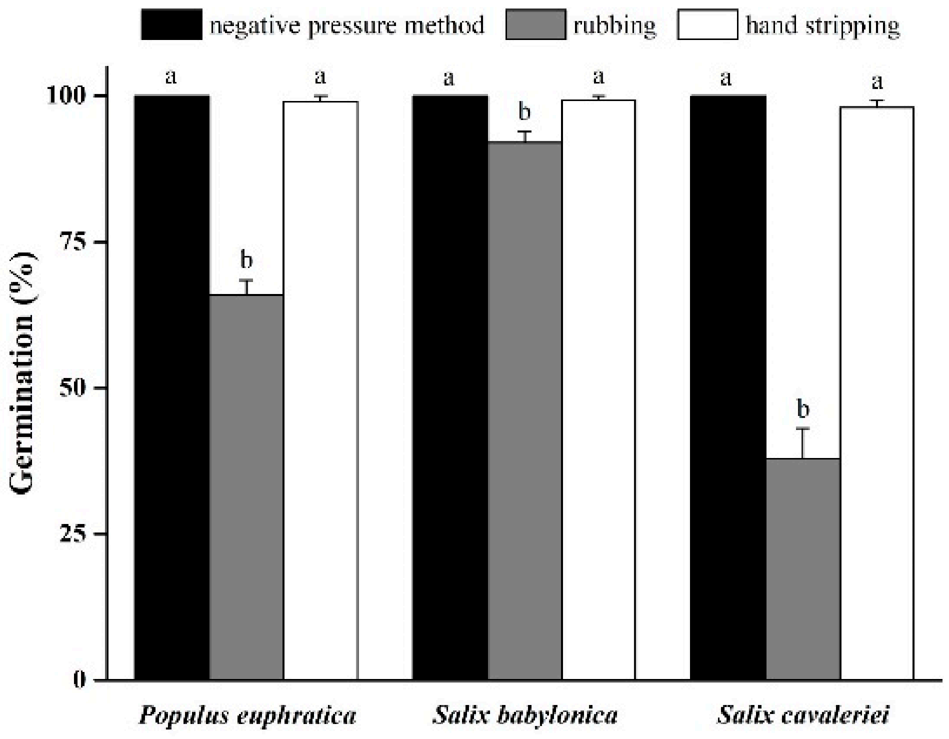

Figure S3. Effect of cleaning method on germination of *Populus euphratica, Salix babylonica* and *Salix cavalerei* seeds. Errors bars indicate SE for three or five replicates of 20 or 50 seeds. Different letters above the bars indicate significant differences ($P < 0.05$) among three clearing methods within each accession.

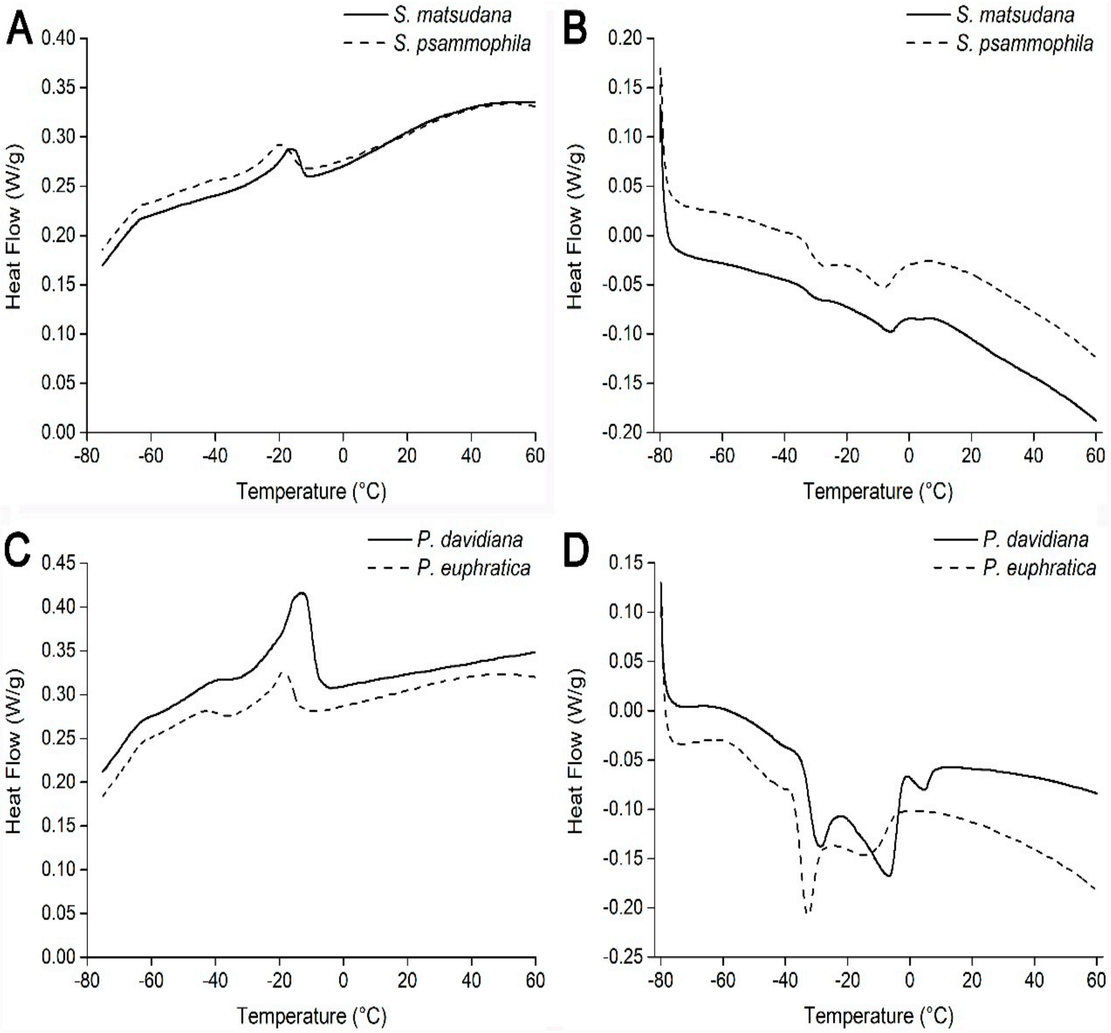

Figure S4. Thermal fingerprints of dry seeds of four Salicaceae species: *Salix matsudana* and *S. psammophila* (A, B) and *Populus davidiana* and *P. euphratica* (C, D). Differential scanning calorimetry thermograms were generated when samples were held at 20 °C for 1 min warmed to 60 °C, held for 1 min and cooled to -80 °C (cooling [B, D]), held for 1 min and re-warmed to 60 °C (warming [A, C]). Cooling and warming were 10 °C per min. Prior to DSC, seeds were aged for 3 d at 20°C and 50% RH (Figure S6).

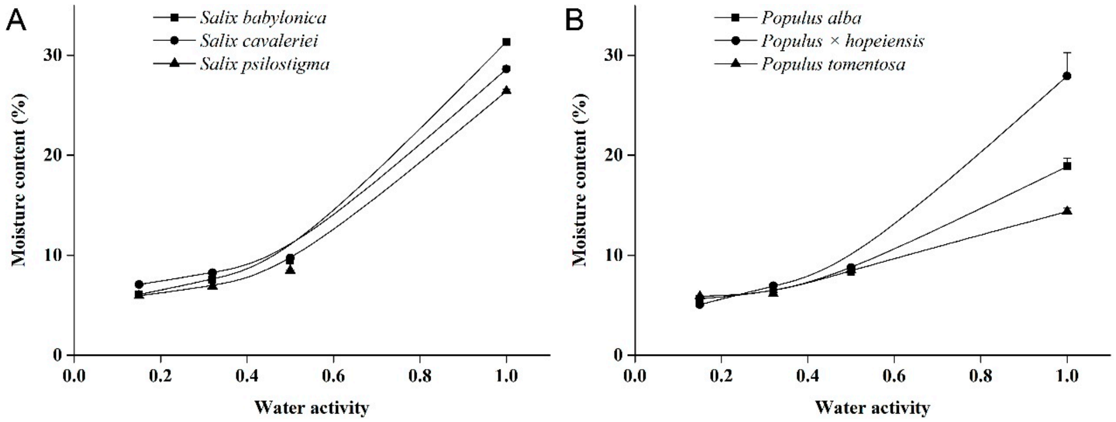

Figure S5. Relationship between seed equilibrium moisture content (MC) and water activity (a_w) at 15 ± 2 ℃, i.e., the water sorption isotherms. (A) Seeds of three *Salix* species seeds: *Salix babylonica* (■) , *Salix cavaleriei* (●) and *Salix psilostigma* (▲). (B) Seeds of three *Populus* species: *Populus alba* (■), *Populus* x *hopeiensis* (●) and *Populus lomentosa* (▲). Values are means of three replicates of 20 seeds ± SE.

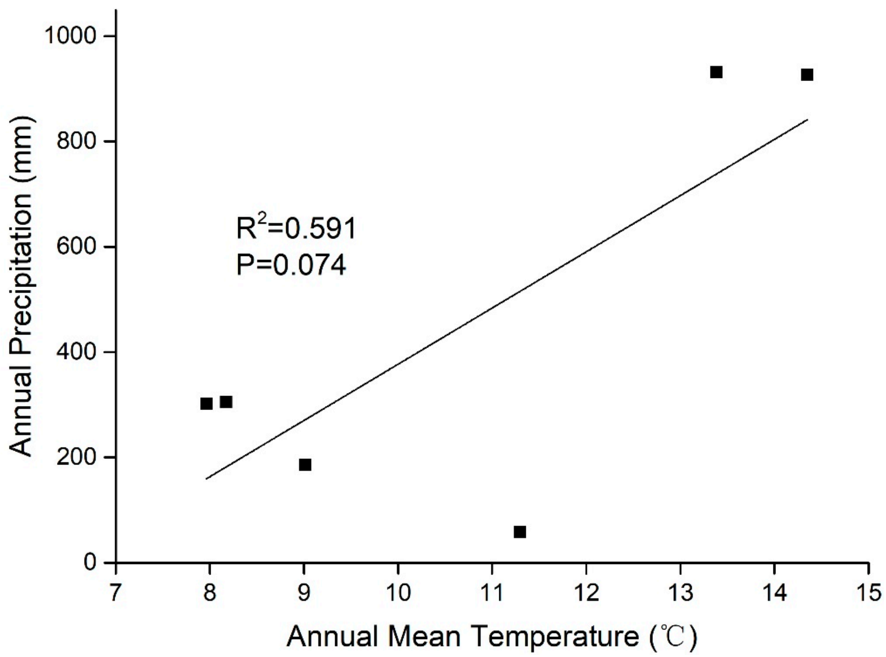

Figure S6. Correlation between annual temperature as well as annual precipitation of the collecting sites of six *Populus* seedlots.

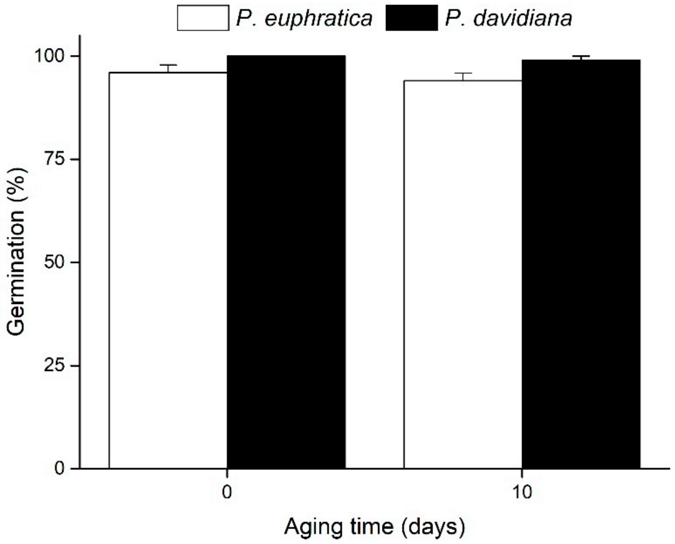

Figure S7. Effect of 10 d ageing on germination of *P. euphratica* (S2) and *P. davidiana* seeds. Seeds were aged at 20 ℃, 50 % RH. Values are means of five replicates of 20 seeds ± SE.

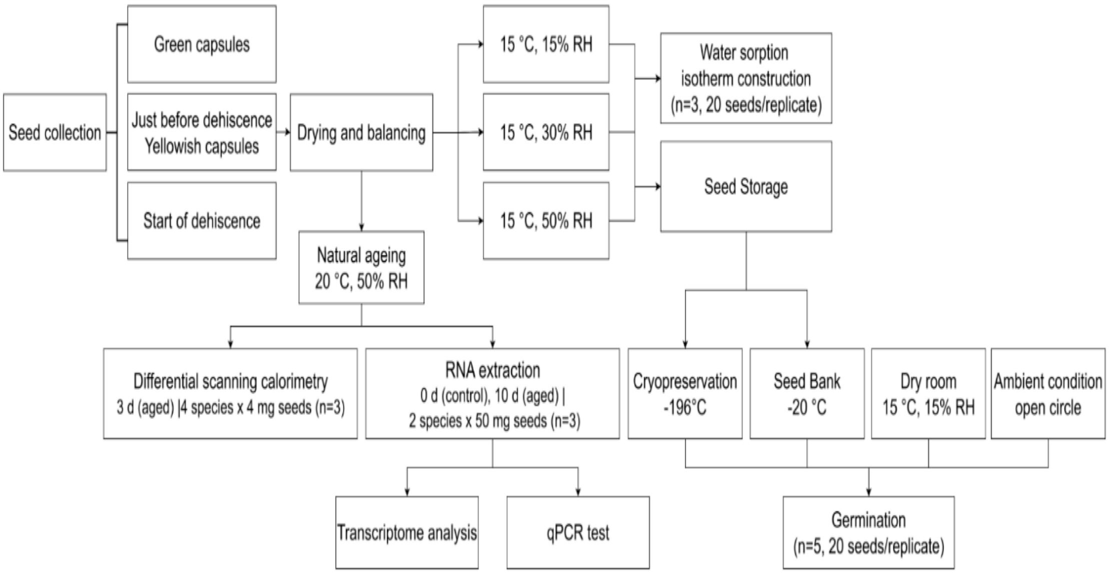

Figure S8. Experimental design and workflow of the seed ageing experiments.

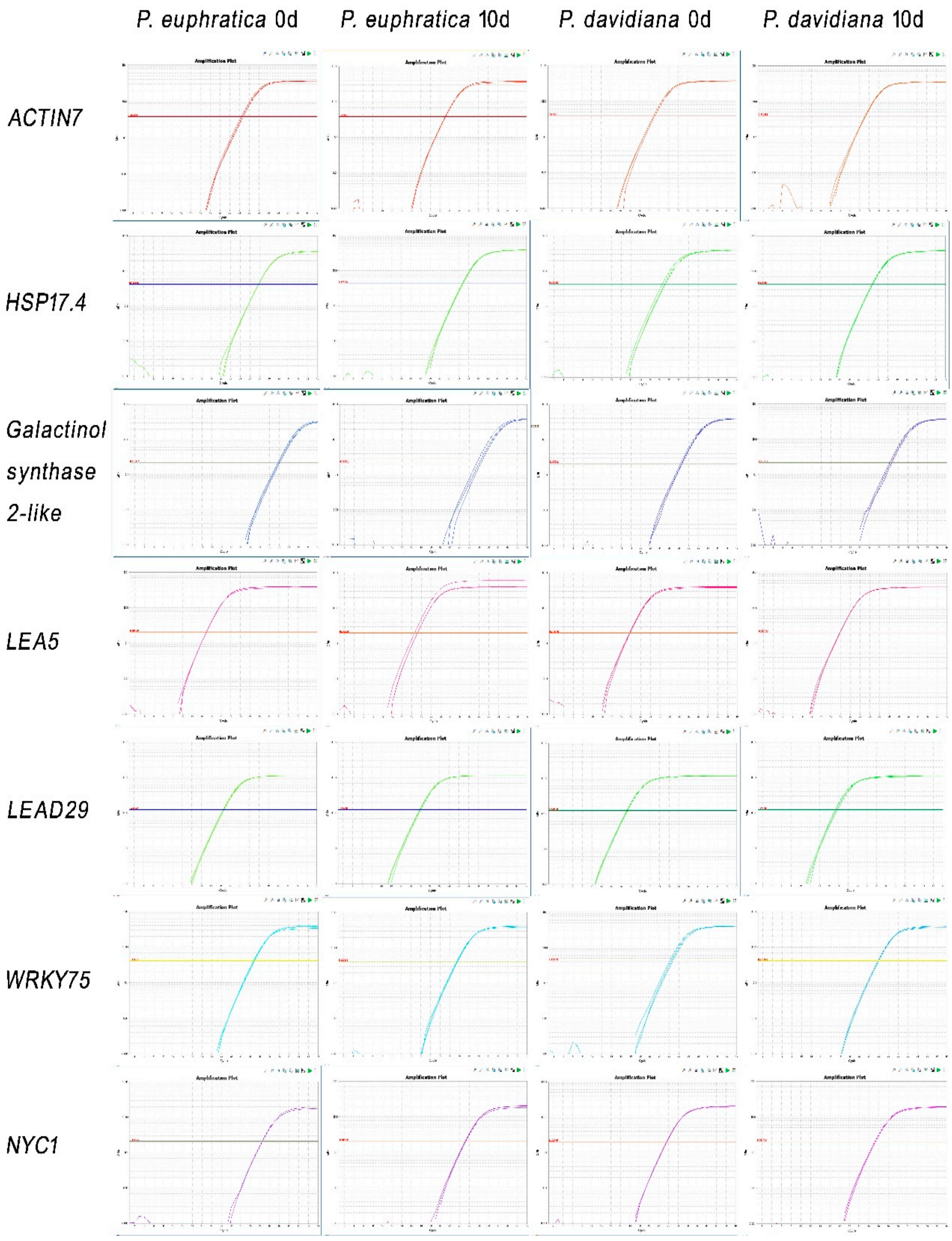

Figure S9. Amplification plot of seven genes during seed ageing process of *P. euphratica* and *P. davidiana*.

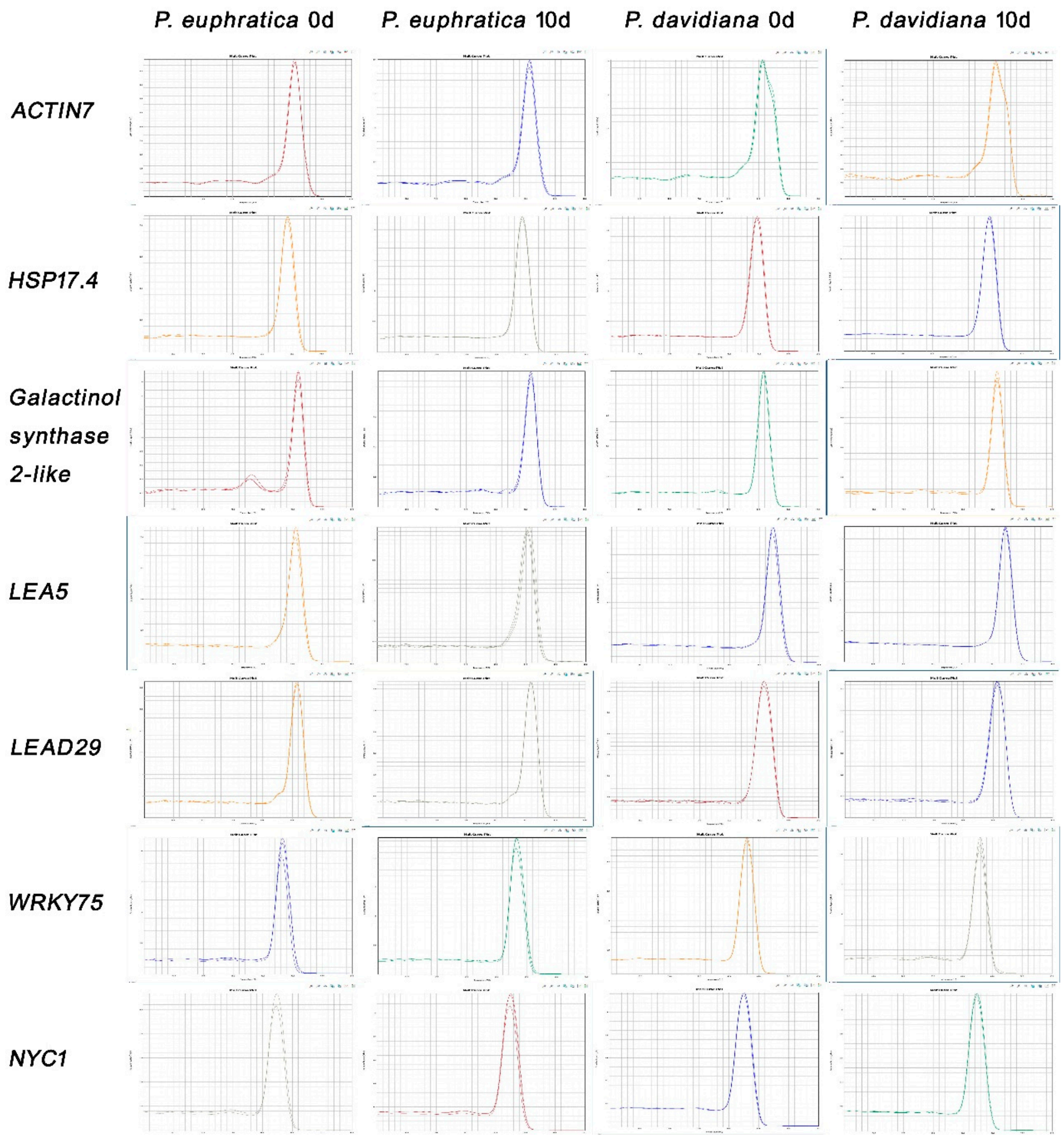

Figure S10. Melting curves of seven genes during the seed ageing process of *P. euphratica* and *P. davidiana*.

Table S1. Primers used for qRT-PCR in this research.

Gene name	Primer	Sequence (5'-3')
HSP17.4	HSP17.4-F	CCTGGCTTGTCCAAGTCTGA
	HSP17.4-R	AGCTTCTGAGGTGCCTTCC
Galactinol synthase 2-like	Gal-F	CGACGGACTTGCAGAAGAGA
	Gal-R	GGCTCAATCTCACGGACGATA
LEA5	LEA5-F	CGCTCTCTCCCAAACGCTAA
	LEA5-R	CCCTACCAAAGCTCGCTGTTA
LEA D29	Pe-LEAD29-F	GAGAAGGCAGAAGAGGATGGTA
	Pe-LEAD29-R	GGTAGTGTCAGAGGCTGTATCA
	Pd-LEAD29-F	CTGATACAGCCTCCGACACT
	Pd-LEAD29-R	CCTGTGGTGGAAGCAATCG
WRKY75	WRKY75-F	GCCGAGTTGATATCCTTGATGA
	WRKY75-R	GTTGTCACCACGACTCCTTC
NYC1	NYC1-F	GCAGTGGCTAGATTGGAGGA
	NYC1-R	AACCAACAGCAAGCACTACTC
ACTIN7	ACTIN7-F	AATGGAACTGGAATGGTGAAGG
	ACTIN7-R	TGCTTACAATACCGTGCTCAAT

Table S2. Quality control of raw reads of *Populus euphratica*. The reference Genome and annotation version is GCF_000495115.2.

Sample	RawDatas	CleanData(%)	Adapter(%)	LowQuality(%)	polyA(%)	N(%)
Euphratica0d1	41009320	40774850 (99.43%)	13774 (0.03%)	218140 (0.53%)	0 (0.00%)	2556 (0.01%)
Euphratica0d2	41320796	41056666 (99.36%)	14452 (0.03%)	246986 (0.60%)	0 (0.00%)	2692 (0.01%)
Euphratica0d3	53277744	52911446 (99.31%)	18694 (0.04%)	343624 (0.64%)	0 (0.00%)	3980 (0.01%)
Euphratica10d1	41604004	41345584 (99.38%)	11054 (0.03%)	246486 (0.59%)	0 (0.00%)	880 (0.00%)
Euphratica10d2	40176372	39924640 (99.37%)	16388 (0.04%)	232676 (0.58%)	0 (0.00%)	2668 (0.01%)
Euphratica10d3	47658092	47450258 (99.56%)	15996 (0.03%)	186686 (0.39%)	0 (0.00%)	5152 (0.01%)

Sample	RawData(bp)	BF_Q20(%)	BF_Q30(%)	BF_N(%)	BF_GC(%)	CleanData(bp)
Euphratica0d1	6151398000	6040980716 (98.21%)	5848763452 (95.08%)	475244 (0.01%)	2728141300 (44.35%)	6053077634
Euphratica0d2	6198119400	6078828729 (98.08%)	5874634664 (94.78%)	484927 (0.01%)	2749644933 (44.36%)	6084274460
Euphratica0d3	7991661600	7811110698 (97.74%)	7487829054 (93.70%)	557206 (0.01%)	3546801817 (44.38%)	7838367481
Euphratica10d1	6240600600	6117516036 (98.03%)	5905281858 (94.63%)	1085188 (0.02%)	2769785255 (44.38%)	6141537900
Euphratica10d2	6026455800	5912762325 (98.11%)	5718911762 (94.90%)	473768 (0.01%)	2676736480 (44.42%)	5926699676
Euphratica10d3	7148713800	7057208767 (98.72%)	6894156065 (96.44%)	722244 (0.01%)	3175981240 (44.43%)	7054856753

CleanData(bp)	AF_Q20(%)	AF_Q30(%)	AF_N(%)	AF_GC(%)
6053077634	5963129501 (98.51%)	5787515444 (95.61%)	430465 (0.01%)	2675923476 (44.21%)
6084274460	5988433849 (98.42%)	5803113205 (95.38%)	437053 (0.01%)	2688667426 (44.19%)
7838367481	7689740238 (98.10%)	7393801759 (94.33%)	486315 (0.01%)	3466261231 (44.22%)
6141537900	6040401358 (98.35%)	5845754834 (95.18%)	1055328 (0.02%)	2717426749 (44.25%)
5926699676	5834824573 (98.45%)	5658108051 (95.47%)	426729 (0.01%)	2623372834 (44.26%)
7054856753	6981150210 (98.96%)	6832208623 (96.84%)	628938 (0.01%)	3125756562 (44.31%)

Sample	Total	Unmapped(%)	Unique_Mapped(%)	Multiple_Mapped(%)	Total_Mapped(%)
Euphratica0d1	40302542	2478248 (6.15%)	35192169 (87.32%)	2632125 (6.53%)	37824294 (93.85%)
Euphratica0d2	40597582	2558936 (6.30%)	35386603 (87.16%)	2652043 (6.53%)	38038646 (93.70%)
Euphratica0d3	52339828	3366839 (6.43%)	45513516 (86.96%)	3459473 (6.61%)	48972989 (93.57%)
Euphratica10d1	40735886	2584337 (6.34%)	35452568 (87.03%)	2698981 (6.63%)	38151549 (93.66%)
Euphratica10d2	39347792	2483756 (6.31%)	34281208 (87.12%)	2582828 (6.56%)	36864036 (93.69%)
Euphratica10d3	46805276	2696600 (5.76%)	40969345 (87.53%)	3139331 (6.71%)	44108676 (94.24%)

Table S3. Quality control of raw reads of Populus davidiana. The reference Genome and annotation version is GCF_000495115.2.

Sample	RawDatas	CleanData(%)	Adapter(%)	LowQuality(%)	polyA(%)	N(%)
Davidiana0d1	49937402	49647982 (99.42%)	16904 (0.03%)	271498 (0.54%)	0 (0.00%)	1018 (0.00%)
Davidiana0d2	45771078	45500850 (99.41%)	11946 (0.03%)	257304 (0.56%)	0 (0.00%)	978 (0.00%)
Davidiana0d3	53323148	52883430 (99.18%)	28876 (0.05%)	406576 (0.76%)	0 (0.00%)	4266 (0.01%)
Davidiana10d1	46426788	46124140 (99.35%)	11482 (0.02%)	290114 (0.62%)	0 (0.00%)	1052 (0.00%)
Davidiana10d2	50777246	50464056 (99.38%)	12352 (0.02%)	299656 (0.59%)	0 (0.00%)	1182 (0.00%)
Davidiana10d3	46097654	45895804 (99.56%)	19344 (0.04%)	177218 (0.38%)	0 (0.00%)	5288 (0.01%)

Sample	RawData(bp)	BF_Q20(%)	BF_Q30(%)	BF_N(%)	BF_GC(%)	CleanData(bp)	
Davidiana0d1	7490610300	7344448379 (98.05%)	7093433120 (94.70%)	1300758 (0.02%)	3334883924 (44.52%)	7357843085	72
Davidiana0d2	6865661700	6732115324 (98.05%)	6502659001 (94.71%)	1193017 (0.02%)	3032703106 (44.17%)	6760525396	66
Davidiana0d3	7998472200	7803072475 (97.56%)	7467976201 (93.37%)	567182 (0.01%)	3553788704 (44.43%)	7808652568	76
Davidiana10d1	6964018200	6823121994 (97.98%)	6584304488 (94.55%)	1214164 (0.02%)	3097784715 (44.48%)	6855943335	67
Davidiana10d2	7616586900	7465132886 (98.01%)	7206245027 (94.61%)	1324503 (0.02%)	3387164616 (44.47%)	7503193944	73
Davidiana10d3	6914648100	6822963139 (98.67%)	6662559939 (96.35%)	698530 (0.01%)	3081391850 (44.56%)	6810441967	67

CleanData(bp)	AF_Q20(%)	AF_Q30(%)	AF_N(%)	AF_GC(%)
7357843085	7239308128 (98.39%)	7010876009 (95.28%)	1263876 (0.02%)	3264827240 (44.37%)
6760525396	6651011774 (98.38%)	6440596744 (95.27%)	1161618 (0.02%)	2976784024 (44.03%)
7808652568	7654559913 (98.03%)	7354123376 (94.18%)	489207 (0.01%)	3454917970 (44.24%)
6855943335	6741469801 (98.33%)	6523377859 (95.15%)	1181445 (0.02%)	3040495834 (44.35%)
7503193944	7378462541 (98.34%)	7140654528 (95.17%)	1287672 (0.02%)	3326803932 (44.34%)
6810441967	6737696319 (98.93%)	6592356838 (96.80%)	602821 (0.01%)	3025021824 (44.42%)

Sample	Total	Unmapped(%)	Unique_Mapped(%)	Multiple_Mapped(%)	Total_Mapped(%)
Davidiana0d1	49197552	5178814 (10.53%)	42487145 (86.36%)	1531593 (3.11%)	44018738 (89.47%)
Davidiana0d2	45094060	4904942 (10.88%)	38788992 (86.02%)	1400126 (3.10%)	40189118 (89.12%)
Davidiana0d3	52424236	5826759 (11.11%)	44953169 (85.75%)	1644308 (3.14%)	46597477 (88.89%)
Davidiana10d1	45621882	4962941 (10.88%)	39206117 (85.94%)	1452824 (3.18%)	40658941 (89.12%)
Davidiana10d2	49913820	5319348 (10.66%)	43004231 (86.16%)	1590241 (3.19%)	44594472 (89.34%)
Davidiana10d3	45396782	4682468 (10.31%)	39258949 (86.48%)	1455365 (3.21%)	40714314 (89.69%)
